# Antisense oligonucleotide inhibition of hepatitis C virus genotype 4 replication in HepG2 cells

**DOI:** 10.1186/1475-2867-6-18

**Published:** 2006-06-27

**Authors:** Mostafa K El Awady, Noha G Badr El Din, Wael T El Garf, Samar S Youssef, Moataza H Omran, Jasmin El Abd, Said A Goueli

**Affiliations:** 1 Department of Biomedical Technology, National Research Center, Dokki; 2 Research and Development, Promega Corp., University of Wisconsin, Madison, USA; 3 Department of Pathology and Laboratory Medicine, University of Wisconsin, Madison, USA

## Abstract

**Background:**

Hepatitis C (HCV) viral infection is a serious medical problem in Egypt and it has a devastating impact on the Egyptian economy. It is estimated that over 15% of Egyptians are infected by the virus and thus finding a cure for this disease is of utmost importance. Current therapies for hepatitis C virus (HCV) genotype 4 with interferon/ribavirin have not been successful and thus the development of alternative therapy for this genotype is disparately needed.

**Results:**

Although previous studies utilizing viral subgenomic or full cDNA fragments linked to reporter genes transfected into adhered cells or in a cell free system showed promise, demonstration of efficient viral replication was lacking. Thus, we utilized HepG2 cells infected with native HCV RNA genomes in a replication competent system and used antisense phosphorothioate Oligonucleotides (S-ODN) against stem loop IIId and the AUG translation start site of the viral polyprotein precursor to monitor viral replication. We were able to show complete arrest of intracellular replication of HCV-4 at 1 uM S-ODN, thus providing a proof of concept for the potential antiviral activity of S-ODN on native genomic replication of HCV genotype 4.

**Conclusion:**

We have successfully demonstrated that by using two S-ODNs [(S-ODN1 (nt 326–348) and S-ODN-2 (nt 264–282)], we were able to completely inhibit viral replication in culture, thus confirming earlier reports on subgenomic constructs and suggesting a potential therapeutic value in HCV type 4.

## 1. Background

It is estimated that over 170 million people are infected globally with hepatitis C virus (HCV), and its devastating impact is further magnified by the high frequency of HCV persistence during infection, i.e., establishing a chronic infection in up to 85% of cases [1]. HCV infection has become the most common cause of hepatocellular carcinoma, and the primary reason for liver transplantations among adults in the western world [2]. There are no broadly effective anti-HCV compounds and therefore new and better therapeutic strategies are desperately needed in the battle against HCV [3]. Several issues that are pertinent to HCV infections made it difficult to develop an effective therapy. These include genetic diversity during replication in the host, development of drug resistant virus mutants, and the lack of reproducible infectious culture systems and small animal models for HCV replication and pathogenesis. Although interferon-α treatment as antiviral therapy has been beneficial, it is limited by the adverse side effects such as flu-like syndrome, and is only successful in 15% of patients [4]. Combination therapy of the more stable, pegylated IFN-α and ribavirin improves response rate to more than 50% with fewer side effects [5]; which makes it the standard treatment for chronic HCV. However, most patients with chronic HCV infection are not candidates for IFN-α-based therapies, and the IFN-α-treatments has limited efficacy in immunocompromised patients and treatment of HCV/HIV co-infection presents another challenge. So the development of alternative therapeutic interventions based on newer strategies is urgently needed. A novel strategy that has emerged in the last few years is to target HCV genomic RNA by using antisense oligonucleotide (ASO) technology, which inhibits gene expression by inducing cleavage of the target RNA at the site of oligonucleotide hybridization by an RNase H-mediated mechanism. Clinical evaluations are underway for the efficacy of ASOs-based drugs in patients with prostate cancer, pancreatic cancer, colorectal cancer, Crohn's disease, rheumatoid arthritis, asthma, HIV-infected patients, etc. [6]. The approval of Vitravene as antisense drug for treatment of cytomegalovirus (CMV)-induced retinitis in AIDS patients paves the way for attempts towards finding an antisense drug that can be successfully used for treatment of HCV infected patients [6]. Several ASOs that have been designed to bind to the stem-loop structures in the HCV Internal Ribosome Entry Sites (IRES) have been effective in inhibiting HCV replication in cell-culture assays and the expression of HCV luciferase reporter gene in the livers of mice infected with recombinant vaccinia virus expressing the reporter construct [7]. Studies on HCV using ASOs have utilized antisense phosphorothioate oligonucleotides (S-ODN) that were designed as complementary to sequences present in the 5' non coding region (5'-NCR) of IRES of the viral genome. These studies were carried out using inhibition of gene expression in HCV-luciferase reporter constructs as a readout, or using inhibition of viral replication using subgenomic HCV containing 5'-NCR, core, and part of the envelope proteins components driven by HCMV immediate early promoter [8]. The use of such subgenomic or genomic replicon has been useful in elucidating the replicative machinery of the virus but could not mimic the actual viral replication cycle and shedding of the virus to the culture medium. Despite the extremely robust in vivo replication rate of HCV, efforts to propagate the virus in cell culture have been frustratingly unsuccessful [9]. Thus the viral replication but not the biologically relevant infectious viral particles can be demonstrated by such an approach.

In the present study we elected to make use of HepG2 cells infected with native viral particles from HCV type 4 positive serum, the most prevalent type in Egypt. We were able to maintain these cells in culture for more than 4 months and they are capable of supporting HCV replication as indicated by consistent synthesis of plus and minus RNA strands by nested RT-PCR and by real-time PCR technique. We show that the two S-ODNs we selected, S-ODN1 (nt 326–348) and S-ODN-2 (nt 264–282), completely inhibited viral replication in culture, thus confirming earlier reports on subgenomic constructs and suggesting a potential therapeutic value in HCV type 4.

.

## 2. Materials and methods

### 2.1 Sequence analyses of 5'UTR in local HCV quasispecies

**Figure 1:**
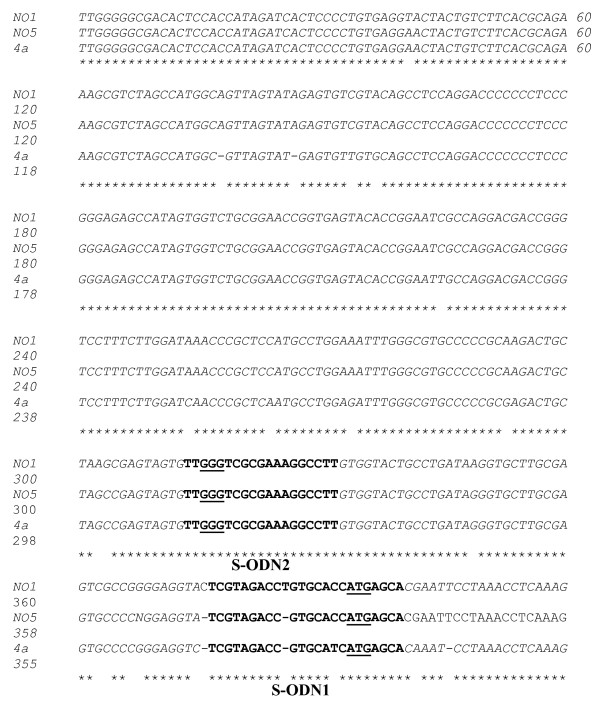
**Nucleotide sequence of 5' UTR in viral quasispecies**. 5' UTR from a pool of HCV infected sera was RT-PCR amplified using primers p1 and P2 and ~340 bp product spanning the entire 5' UTR was cloned into pGEM-T plasmid. Single colony from transformed JM109 cells were used for plasmid DNA purification and sequencing. NO1 and NO2 are representative clones from 17 isolates of 5' UTR fragments. The bold sequences represent targets for antisense phosphothioate oligonucleotides (S-ODN1 and S-ODN2). Highly conserved triplets necessary for efficient initiation of translation are shown in bold underlined letters.

Serum samples were collected from five HCV positive patients who were diagnosed by detectable HCV RNA using nested RT-PCR method as described [10]. RNA samples were extracted and the entire 5' UTR was reverse transcribed using P2 as 3' end primer and then amplified using P1 as forward primer and P2 as the reverse primer. Successful amplification was confirmed by employing a nested amplification using primers P3 and P4. Five to ten first round amplification products from each patient were collected and the 340 bp DNA generated fragment was ligated into pGEM-T plasmid (Promega Madison, WI) and transformed into competent JM109 *E. Coli*. Seventeen recombinant plasmids were purified from individual white colonies using mini preparation method (Promega, Madison, WI). Insert DNA clones were sequenced in both the forward and the reverse directions using Sp6 and T7 primers respectively. Cycle sequencing reactions were performed using the Big Dye terminator method (ABI Foster City, CA). The sequence of each quasispecies was determined on ABI 310 prism (ABI Foster City, CA). The sequences obtained from 17 5' UTR fragments, each representing independent isolate, were aligned with the published sequence from HCV genotype, 4a. Alignments of only two local HCV isolates with type 4a are shown in figure 1.

### 2.2 Design and synthesis of oligodeoxynucleotides

The highly conserved regions among all HCV isolates were identified as targets for antisense (S-ODN). Based on earlier studies [8,11] two IRES motifs represented targets for the most efficient inhibition of viral replication by ODN namely; S-ODN1 (nt 316 – 339) and S-ODN2 (nt 254 – 272) as in fig 1.

Although alignment of 5' UTR sequences in type 4a had nucleotide differences ranging from 3.5% to 5.3% when compared to isolates of type 4 used in the present study, the two stem loop targets for S-ODN1 and S-ODN2 are conserved among all isolates analyzed except for a single mismatch in only one isolate (NO1). Therefore, we selected the following two sequences for S-ODN design

S-ODN1 (5'TGCTCATGGTGCACGGTCTACGA3');

S-ODN2 (5' GGCCTTTCGCGACCCAA 3').

Antisense nucleotides were purchased from biognostik, Gesellschaft fur molekulare diagnostik, Gottingen (Germany). Phosphorothioate DNA were synthesized as Na -salts and systematically purified using 2 steps high pressure liquid chromatography followed by cation exchange chromatography, and sterile ultra-filtration to remove any interfering substances that might be toxic to culture systems.

### 2.3 HepG2 cell culture and infection with HCV

HepG2 cells were obtained from the American Type Culture Collection (ATCC HB8065) and maintained in 75 cm^2 ^tissue culture flasks (Greiner bio-one GmbH, Germany) containing Dulbecco's Modified Eagle's Medium with 0.45 % Glucose and 1 % L-Glutamine (DMEM; BioWhittaker, Combrex Company, Belgium) supplemented with 10 % Fetal Calf Serum (FCS; Biochrome KG Berlin Germany) and antibiotics (penicillin/streptomycin 10000 μ/10000 μg/ml (Biochrome KG Berlin Germany) and fungisone (GIBCO- BRL life technologies, Grand Island NY). The cells were fed fresh medium every 3 days and were grown to semi-confluence (8 to 10 days) and were then sub-cultured.

The principal inoculum was a serum sample obtained from a 23 year old male patient, who was positive for anti-HCV antibodies and HCV RNA. HCV genotype in this sample was identified as type 4 using the method described by Ohno et al [12]. Sequence analysis of 5'UTR in three isolates cloned from this patient revealed significant homology to the published strains and 100 % sequence conservation at IRES stem loop structures. Viral load in the serum sample used was 290,000 copies/ml. Cells were maintained in complete medium (8 ml) for 48 hours at 37°C. Cell layers were washed twice with FCS-free medium and incubated with 500 μl HCV positive serum plus 500 μl FCS-free DMEM for 90 minutes. Medium and FCS were then added to make a final of 10 % FCS in 8 ml complete DMEM. The cells were maintained overnight at 37°C in 5 % CO_2_. Next day, adherent cells were washed three times with culture medium and incubation continued in complete medium supplemented with 10 % FCS with regular medium changes. Assessment of the viral infection in HepG2 cells throughout the culture duration was confirmed by RT-PCR amplification of plus and minus strand as described previously (13) as well as consistent viral load by real time PCR over 4 month period in culture. S-ODN1 and S-ODN2 were added to infected cells in culture wells (3 wells for each treatment) at 1 μM and 2 μM and were maintained for 24, 48 and 72 hours.

### 2.4 Detection of plus-, and minus-strand RNA by nested RT-PCR

*C*ellular RNA's from three separate wells were extracted using SEEK VIRAL RNA extraction kit (TALENT, Trieste-Italy) and subjected to nested RT-PCR analysis. Total RNA from cultured HepG2 cells were reverse transcribed and amplified using primer sequences derived from the highly conserved noncoding region of HCV genome as described [10]. The reaction was performed in 25 ul reaction mixture containing 20 units of AMV reverse transcriptase (Clonetech, USA), 200–400 ng of total cellular RNA as template, 40 units of RNAsin (Clonetech, USA), 0.2 mmol/l from each dNTP (QBIOGENE, USA), and 50 pmol of the reverse primer p2 (for plus strand) or 50 pmol of the forward primer p1 (for minus strand). The reaction was incubated at 42°C for 60 min. and denatured at 98°C for 10 min. Amplification of the highly conserved 5'-UTR sequences was done using two rounds of PCR with 2 pairs of nested primers. First round amplification was done in 50 ul reaction containing 50 pmol from each of p1 forward primer and P2 reverse primer, 0.2 mmol/l from each dNTP, 10 ul from RT reaction mixture as template and 2 units of Taq DNA polymerase (Finnzyme, USA) in a 1× buffer supplied with the enzyme. The thermal cycling protocol was as follows: 1 min. at 94°C, 1 min at 55°C and 1 min at 72°C for 30 cycles. The second round of amplification was done similar to the first round, except for use of the nested reverse primer p4 and forward primer p3 at 50 pmol each. A fragment of 171 bp length was identified in positive samples. Primer sequences were as follows:

P1 5'AACTACTGTCTTCACGCAGAA 3'

P2 5' GGTGCACGGTCTACGAGACCTC 3'

P3 5' GTGCAGCCTCCAGGACCC 3'

P4 5' ACTCGGCTAGCAGTCTCGCG 3'

### 2.5 RNA quantification of in-vitro infection

#### HCV RNA quantification of in-vitro infection

Plus-strand RNA was transcribed in-vitro from a cloned fragment of the HCV genome encompassing the entire 5' UTR in pGEM-T plasmid using in vitro transcription system as described by the manufacturer (Promega, Madison, WI, unpublished data). The transcribed 5' UTR RNA was purified and quantified by O.D_260_. Serial copy numbers ranging from 2 × 10^6 ^– 2 × 10^7 ^copies/reaction were reverse transcribed and amplified using the same RT-PCR primers and same protocol described above for plus strand amplification. Amplified products from nested RT-PCR reactions of RNA isolated from infected cells and standards were resolved on 2% agarose gel and stained with ethidium bromide. Polaroid photographed gels were scanned and the intensity of the amplified bands were analyzed using Total Lab software (Phoretix, Newcastle, UK). Numbers of copies per each 5 ÙTR concentrations were plotted against number of intensity units expressed as pixels. The number of copies in each specimen was calculated on the standard curve using the number of pixels in each case

#### Quantification of human Glyceraldehyde-3-phosphate dehydrogenase (GAPDH) mRNA

To check the integrity of the cellular RNA preparations from HCV infected HepG2 cells, we quantified GAPDH mRNA in the absence and in presence of S-ODN1 and S-ODN2. We wanted to ensure that S-ODN used in this study do not adversely affect the expression of a house keeping gene from host cells. The GAPDH mRNA levels were quantified by real time RT-PCR using TaqMan technology and GAPDH specific primers [14]. Amplification of human GAPDH transcripts was performed basically using the TaqMan EZ RT-PCR kit (Applied Biosystems, Foster City, CA). The target template was the purified cellular RNA from HepG2 cells at 24 and 48 hours post infection with HCV, in absence and presence of S-ODN1 or S-ODN2 (at either 1 μM or 2 μM each). Reverse transcription-PCR was done by using a single-tube, single-enzyme system. The reaction exploits the 5'-nuclease activity of the rTth DNA polymerase to cleave a TaqMan fluorogenic probe that anneals to the cDNA, during PCR, between the forward primer at nucleotide position 1457 and reverse primer at nucleotide position 3412 of the human GAPDH gene. In a 50 μl reaction volume, 1.5 μl of RNA template solution equivalent to total cellular RNA from 2.5 × 10^5 ^cells were mixed with 200 nM forward primer, 100 nM reverse primer, 100 nM GAPDH probe, 300 μM from each of dATP, dCTP, dGTP and 600 uM dUTP, 3 mM manganese acetate, 0.5 u rTth DNA polymerase, 0.5 u Amp Erase UNG, 1× Taqman EZ buffer and amplified in the sequence detection system ABI 7700 (Applied Biosystems, Foster City, CA). The RT-PCR thermal protocol was as follows: Initial UNG treatment at 50°C for 2', reverse transcription at 60°C for 30', deactivation of UNG at 95°C for 5' followed by 40 cycles each consists of denaturation at 94°C for 20" and annealing/extension at 62°C for 1'.

### 2.6 Statistical analysis

**Figure 2:**
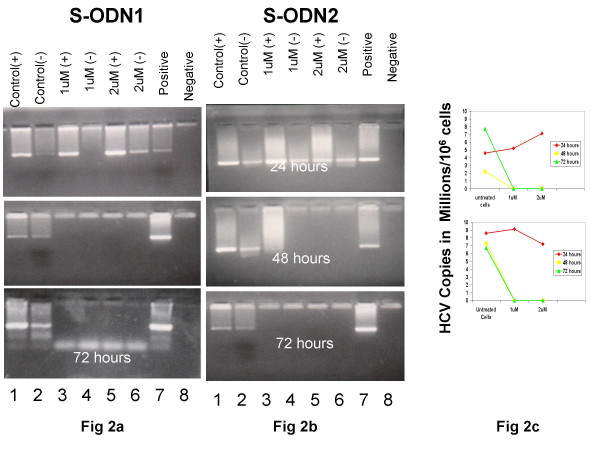
**Inhibition of intracellular plus and minus-RNA strands by antisense primers**. HepG2 cells were cultured in absence (lanes 1,2) and presence of S-ODN1 (Fig 2a) or S-ODN2 (Fig 2b) at either 1 μM (lanes 3,4) or 2 μM (lanes 5,6). Cellular RNA was reverse transcribed using plus strand (lanes 1,3,5) or minus-strand (lanes 2,4,6) specific primers. cDNAs were amplified by nested PCR as in materials and methods. RNA from infected and uninfected sera were similarly amplified to serve as positive (lane7) and negative (lane 8) controls. Band density in each lane was scanned and measured using Total-Lab software. Relative viral copies/cell are represented in figure 2c.

**Figure 3:**
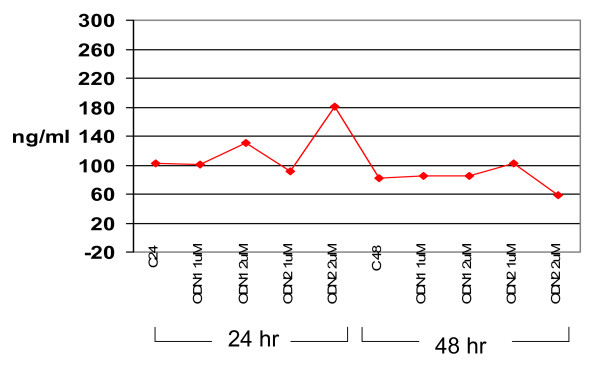
**Concentration of GAPDH transcripts in HepG2 cells treated with antisense primers**. HepG2 cells were cultured in absence and in presence of S-ODN1 and S-ODN2 at concentrations of 1 μM or 2 μM for 24 h. & 48 h. as described in legend for fig(2). Purified cellular RNA from 2.5 × 10^5 ^cells was RT-PCR amplified using the Taqman EZ-RT-PCR kit as in materials and methods. Fluorescent-labeled amplicons from different samples was calculated for each sample from standard GAPDH RNA provided with kit, the concentrations of GAPDH transcripts throughout the culture were plotted as lines.

The data sown in Figure 2 and 3 were carried out at least in triplicates for each treatment and data averages with standard errors of the means are shown.

## 3. Results

### Identification of sequence conservation among IRES

To determine the conservation of IRES motifs across HCV isolates, nucleotide sequences of 17 5'NCR clones from 5 HCV type 4 infected subjects were analyzed and compared with published 5' UTR. Comparisons of only two isolates (NO1 and NO2) having the greatest sequence diversity within 5'NCR from that of genotype 4a are shown in Fig 1. Nucleotide sequences show identity with genotype 4a except for 13 and 22 nucleotides for NO1 and NO5, respectively while the mismatches between the two isolates is 10 nucleotides. Figure 1 also shows well-conserved blocks in the IRES motifs (1 to 4) and a phylogenetic conservation of triplet GGG (nt 256–258) in stem loop IIId and triplet ATG (nt 333–335) at the translational initiation of the core gene. Based on previous reports, IRES1 and IRES2 spanning the regions (nt 316–338) and (nt 254–272) termed S-ODN1 and S-ODN2 respectively, were predicted to be the best targets for demonstrating inhibitory effects of S-ODN on viral replication and polyprotein expression.

### Effect of S-ODN on the detection of plus- and minus-strand RNA in HepG2 cells

HepG2 cells infected with HCV type 4 were grown in culture for 24, 48 and 72 hours in the absence and presence of two concentrations of antisense S-ODN (1 μM and 2 μM), and the results of nested RT-PCR for HCV plus and minus strand RNA in the absence and presence of S-ODN1 are shown in Fig 2a. It is apparent that both plus-, and minus-RNA strands were detected in HepG2 cells after 24, 48 and 72 hours in culture (control + & -). Addition of S-ODN1 to the culture failed to inhibit plus strand RNA after 24 hours at either concentrations (1 μM and 2 μM). Only the minus-strand RNA was inhibited at 24 hours after addition of 1 μM S-ODN1 to the culture. Both concentrations used for S-ODN1 inhibited completely (100% inhibition) the synthesis of both plus- and minus-strands RNA of HCV after 48 hours and their effects extended till 72 hours of culture. Figure 2b demonstrates the effect of S-ODN2 on plus- and minus-strand RNA of HCV in infected cells. The results shown in this figure are fairly similar to those shown in figure 2a, except that both plus- and minus-RNA strands were detectable throughout the first 24 hours of culture supplemented with either concentration of S-ODN2. Furthermore, plus-strand RNA of HCV was still detectable; although weakly, in presence of 1 μM S-ODN2 after 48 hours. Similar to S-ODN1 (fig. 2a), S-ODN2 completely inhibited the synthesis of both plus and minus RNA strands at 2 μM concentration after 48 hours and at both concentrations after 72 hours (fig. 2b).

### Quantification of the inhibitory effect of S-ODN on intracellular viral load

To quantitatively analyze the inhibitory effect of S-ODN on HCV replication, total cellular RNA was examined for viral copy number in infected cells. Quantification of intracellular plus-strand RNA was performed as described in materials and methods. Figure 2c displays the HCV copy number in infected cells in absence and in presence of S-ODN1 or S-ODN2 in culture for 24, 48, and 72 hr duration. It appears that the viral copy number fluctuates mildly throughout the experiments, ranging from (8 to 10) × 10^3 ^genome equivalents per 10^6 ^cells. When either S-ODN1or S-ODN2 was added at 1 μM or 2 μM concentrations to the culture, no significant changes in viral genome numbers were noted indicating that 24 hrs is insufficient to observe detectable inhibition on HCV load. Whereas, approximately 400 HCV genome equivalents per 10^6 ^cells representing 5% of the initial copy numbers were still amplifiable after 48 hrs in cells treated with 1 μM S-ODN1 whereas 2 μM S-ODN1 totally abolished viral RNA after 48 hrs. Total viral eradication was observed after 48 hrs when cells were stimulated with S-ODN2. In general similar results were obtained after 72 hrs when 1 or 2 μM of either antisense was tested. This indicates that at least a period of 48 hrs is required for either antisense deoxynucleotide to have 100% inhibitory effect on HCV translation driven from the AUG start codon (nt 326–348) and stem loop IIId (nt 264–282).

### Effect of S-ODN1 nucleotides on GAPDH RNA

To demonstrate that the observed inhibitory effect of S-ODN was specific to HCV gene expression, the same cellular RNA samples from HCV/HepG2 cells treated with S-ODN were utilized to amplify the human cellular Glyceraldehyde-3-phosphate dehydrogenase (GAPDH) mRNA using real time PCR and Taqman technology. The results displayed in figure 3 demonstrated that total cellular RNA's used as template for HCV amplification were intact in all preparations used in these experiments and varies within a range of one to two fold increase when compared with GAPDH abundance in control cells. These results clearly demonstrate that the inhibitory effect of antisense S-ODN is specific to HCV translation and the in vitro system described herein. Collectively, these results support the usefulness of HCV consistent replication and testing of antisense S-ODN molecules for their antiviral activity.

## 4. Discussion

The current strategies for treatment of HCV liver disease are not yet satisfactory to the majority of HCV patients. Sustained viral response to interferon α_2 _plus ribavirin combined therapy has been successful for only 10% among Egyptian HCV patients who are predominantly infected with genotype 4 [10]. Data on the use of pegylated interferon in Egyptian patients infected with this HCV genotype have not yet been completed. Moreover, combination therapy has significant side effects and is poorly tolerated by individuals who are affected by other diseases, and the overall chances for a cure are less than 50%. Thus the development of alternative antiviral therapies is of paramount interest to many investigators and clinicians who are dealing with this devastating disease in Egypt. Unlike nonspecific antiviral treatment with interferon-α and ribavirin, target specific antiviral therapy would directly block viral replication and prevent continuing infection of liver. These potential therapies include nucleoside analogues [14], bridged nucleic acids (BNA) [15], inhibitors of viral proteases, helicases and polymerases [16-18], and antisense phosphorothioate oligodeoxynucleotides (S-ODN). The latest therapeutic option i.e. S-ODN has received much attention from several investigators around the world [8,11]. However, as alluded to earlier, the lack of a reliable cell culture system allowing persistent in vitro virus propagation is still hampering screening of antiviral activity of these molecules and the development of effective therapies. Much of the struggle against HCV is caused by its genetically heterogeneous nature and the existence of quasispecies. Quasispecies are distinct but closely related variants of the virus and circulate in the infected individuals. This viral heterogeneity results from high error rate of NS5B gene-coded RNA-dependent RNA polymerase Because the liver is the main target for replication of HCV in vivo, the majority of cell types used for HCV replication in vitro were of a human hepatocyte origin; including human hepatoma, HuH7 [19]; hepatoblastoma, HepG2 [19,20], fetal hepatocytes [21,22] or fused primary human hepatocytes with hepatoblastoma cells [22]. In the present study, the reasons why we utilized HepG2 cells for HCV replication in vitro experiments are attributed to their similarities to primary human hepatocytes in their biosynthetic pathways. An additional advantage of HepG2 cells is the presence of a 66 K Da receptor protein for S-ODN that was purified from HepG2 cell membrane [23], thus allowing reasonable uptake and cytosolic transfer of S-ODN in these cells. There have been several approaches for testing the efficacy of antiviral agents on HCV replication. Transfection of subgenomic viral cDNA fragments that were linked to reporter genes such as the firefly luciferase gene and expressing non structural and structural proteins in various expression systems have been reported [8,11,24]. These viral constructs were not permissible for HCV replication. Alternatively, full-length cDNA clones were constructed from positive stranded viral RNA genomes and were found infectious to cells [25,26]. The viral RNA, produced presumably via transcription of transfected cDNA, is expected to be inactivated due to splicing and polyadenylation processes similar to all nuclear transcripts. Furthermore, a major problem that makes this approach suboptimal for the present study is its structural limitations in terms of the correct length and sequences at 3' and 5' ends of RNA molecules. The later comprises the components of the IRES, which is the main target for S-ODN in this study. The IRES is a highly structured RNA element that directs cap-independent translation of the HCV polyprotein from the 5' end of the plus strand RNA. Although the minus strand 3'-terminal region has the antisequence of the 5'-end of the plus strand, it doesn't fold into its mirror image [27]. Several laboratories have shown that the 3'-terminal sequences of either strand RNA contributes essential biological functions for viral replication [27-29]. We, therefore, hypothesized that the use of viral constructs from cDNA or viral RNA in transfection experiments will deprive the viral replication machinery from the action of host cellular factors, like polypyrimidine-tract binding protein, PTB [30,31] that was found to bind a cis acting element at the 3'end of HCV for viral replication. In the present study, an alternative in vitro system to test S-ODN antiviral function was made by utilization of a well defined HCV inoculum from positive serum in infection experiments to HepG2 cells. Native viral RNA genomes containing the IRES components at the 5' end for efficient translation of viral polyprotein precursor and intact PTB binding elements at the 3' end of the HCV genome for efficient viral replication were expected to provide a fairly natural intracellular system for HCV proliferation. Several reports have shown that the infection experiments in a variety of cells in culture were associated with transient viral replication and minimal viral yield, the in vitro system we describe here has been associated with moderate viral load ~10^4 ^viral copies per 10^6 ^HepG2 cells and prolonged viral replication as well as core and E1 expression for up to 130 days. Furthermore, culture media of these infected cells were found to be highly infectious to naïve cells (results not shown), indicating successful shedding out of infectious viral particles in cell surroundings.

Wide variability in the inhibitory potency of antisense S-ODN targeted against several viral sequences has been reported in a variety of in-vitro systems. The reasons for the limited success with the use of S-ODN against specific stem loop structures, particularly those constituting the IRES elements, is that some of these stem loops form a very stable secondary structures, so that the target motifs for S-ODN contain up to 75% paired RNA nucleotides [8,32] which may interfere with the inhibitory effect of S-ODN. Furthermore, the biological significance of certain stem loops in HCV translation is still not well understood. Site directed mutagenesis of stem loop1 has been previously shown that sequence conservation within this region is not essential for IRES activity [14]. In contrast, stem loop IIId (264–282) was shown to contain the conserved sequence, GGG triplet, which is essential for proper IRES folding [33] and viral translation from the AUG start site located at a distance. In the present study S-ODN structures were designed against the two phylogenetically conserved regions; the region comprising the AUG start codon (S-ODN1) and stem loop IIId (S-ODN2). The sequence data from local isolates revealed conservation at specific motifs related to proper folding and efficient translation i.e. IIId GGG (nucleotide 266–268) and AUG start codon (nucleotide 340–342) respectively. These data offer an advantage for antisense drugs to be a therapeutic option for most known genotypes of HCV. Earlier studies showed that IRES motifs were efficient targets for S-ODNs on constructs containing the 5'-UTR alone or with subgenomic fragments of the virus linked to luciferase reporter in either cell-free system [8] or HepG2 cells [8,11,24]. Our results using cells infected with native viral genome proved to be very sensitive for testing S-ODN inhibitory activity on viral translation/replication. The antisense S-ODN1 and S-ODN2 completely inhibited viral replication at concentrations as low as 1 μM, whereas the use of subgenomic construct in reticulocyte lysate showed inhibition of translation at > 4 μM concentration of the same S-ODN structures [8] The present in-vitro system is advantageous in the sense that the use of higher concentration of S-ODN tend to be nonspecific for translation inhibition. The reason why the use of cell culture provides more sensitivity for S-ODN concentrations than cell-free systems in this and in another study (8) is related to the triggering of the intracellular RNAse H activity by the readily formed RNA-DNA hybrid between viral 5'-UTR RNA and S-ODN DNA molecules, a mechanism that facilitates elimination of viral RNA by RNAse H degradation.

In the current studies we have not examined the specificity of S-ODN_1_and S-ODN_2 _on other viruses. A more reasonable approach to understand specificity of these molecules is to study their influence on expression of human constitutive genes such as GAPDH. Our results indicated that the S-ODN molecules under study are HCV specific with no detectable inhibition on GAPDH mRNA levels. To ensure biological safety of these molecules, studies of the effect of these S-ODNs on other key genes such as those involved in cell cycle and other major signaling pathways need to be evaluated.

Recent studies focused on the use of RNA interference (RNAi) as a new strategy against HCV showed similar success to the antisense oligodeoxynucleotides treatment in inhibiting viral replication in cell culture [34,35]. However for use of RNAi strategy in human patients several major issues have to be addressed. These include poor stability of dsRNA in circulation, dsRNA-induced interferon response resulting in shutting down general protein synthesis, off-target effects of dsRNA, and because of the exquisite sensitivity of RNAi strategy, generation of resistant viruses (escape viruses) due to a single nucleotide change in the target region [36]. Thus, we believe that antisense strategy is more promising in combating HCV.

In summary, the results described in the present in-vitro system indicated that S-ODN1 has relatively more inhibitory potency than S-ODN2, a finding that supports earlier reports [30]. The results described herein also demonstrate the establishment of an in vitro model for the replication of HCV Type 4; a major accomplishment in studying HCV which may facilitates the development of anti HCV therapeutics. Finally, the results also provide evidence that antisense phosphorothioate oligonucleotides targeting stem loop IIId and AUG translation initiation site are effective inhibitors for viral replication and represent potential prototype for treatment of HCV type 4 in liver pathology. Future direction will make use of enhanced delivery strategy of the antisense oligodeoxynucleotides by conjugation to arginine-rich peptides [37]. We have successfully used such an approach to show specific inhibition of growth factor (EGF) as well as phorbol ester-mediated activation of MAP kinase and its phosphorylation of the transcription factor ELK in the nucleus [38]. Such approach may enhance the efficacy of the antisense strategy in mediating the inhibition of HCV replication and thus eliminating the HCV as a dreadful disease that is devastating the Egyptian population.
